# New insights into the milk quality at varying altitudes in China

**DOI:** 10.1016/j.fochx.2024.101492

**Published:** 2024-05-19

**Authors:** Wentao Qian, Xiaobing Wang, Hongliang Li, Yinhua Zhu, Pengjie Wang, Xiaolu Geng, Jinhui Yang, Huiyuan Guo, Menghui Wang, Chong Chen

**Affiliations:** a College of Food Science & Nutritional Engineering, China Agricultural University, Beijing 100083, China; b Beijing Advanced Innovation Center for Food Nutrition and Human Health, Department of Nutrition and Health, China Agricultural University, Beijing 100193, China; c Mengniu Hi-Tech Dairy Products (Beijing) Co., Ltd., Beijing 101100, China; d Inner Mongolia Mengniu Dairy (Group) Co., Ltd., Hohhot 011500, China

**Keywords:** Altitude, Plasmin activity, Milk composition, Holstein, Pasteurization

## Abstract

Introducing Holstein cows on Qinghai-Tibetan Plateau is a potential solution to enhance local milk production. However, the relationship between milk quality and altitude in China remains unknown. Therefore, the components and plasmin (PL) system of raw milk from different altitudes (sea level, 1600, 2700, and 3800 m) were investigated. The daily milk production of Holstein cows and PL activity decreased as the altitude increased. However, the components content of raw milk, plasminogen (PLG)/PL ratio, activities of PLG and plasmin activator (PA) increased with altitude. The pasteurization resulted a significant decrease in PA activity of all milk and a significant increase in PL activity in milk collected at higher altitudes (2700 and 3800 m), suggesting the pasteurization was unsuitable for preserving milk at higher altitudes. This study offered references for the production and storage of milk after introducing Holstein cows on Qinghai-Tibetan Plateau.

## Highlights

• higher altitude showed higher milk composition content and lower daily production.
• The PL activity decreased at increasing elevations.
• The activities of PLG and PA, and PLG/PL ratio increased at higher altitudes.
• The pasteurization might be not suitable for preserving milk from higher altitudes.

## 1 Introduction

Qinghai-Tibetan Plateau is a severe cold and hypoxia area due to its high-altitude conditions (Cui et al., 2016).Yaks are indigenous to the Qinghai-Tibetan Plateau, which have adapted to the prevailing conditions and provided the herders with milk for consumption and livelihoods (Luo et al., 2020). However, the low milk production capacity of yaks (0.9–2.6 kg/day) limits the local supply and demand for dairy products in this region (Singh, Arora, & Sarkar, 2023). Thus, enhancing milk production to satisfy local market requirement is an important issue. Introducing high-yield breeds, such as Holstein cows, on Qinghai-Tibetan Plateau is an potential way to solve the problem. However, the influence of high-altitude conditions on the quality of milk remains unknown.

As Holstein cows usually live in a mild temperate maritime climate, a critical consideration in the introduction is the ability of Holstein cows to sustain the high production of good-quality milk in the challenging climate of Qinghai-Tibetan Plateau. It has been reported that there is a high correlation between the environmental adaptability of Holstein cows and their milk production (Tahir Usman, Ying, & Wang, 2013). The environmental factors, including air temperature, significantly affect the yield and composition of milk from Holstein cows, showing the negative correlations between air temperature and daily milk production, milk fat, and milk protein content (Mylostyvyi & Chernenko, 2019). Furthermore, Vaculíkova et al. reported the adverse effects of decreasing temperatures on Holstein milk yield within the range of −5-10 °C, which closely paralleled the annual average temperature on the Qinghai-Tibetan Plateau (−6–15 °C) (Vaculikova, Komzakova, & Chladek, 2017). Pathak et al. reported significant variations in milk composition with altitude, observing an increase in fat content and a reduction in total solids in the milk from Badir cows at altitudes ranging from 550 to 2084 m (Pathak Abhishek, Shahi, Verma, & Ur, 2021). However, limited researches have specifically investigated the impact of altitude on the milk production of Holstein cows or its composition, particularly in the context of the extremely high altitudes of the Qinghai-Tibetan Plateau. Clarifying the relationship between altitude and milk quality is crucial for dairy-farming practices in this unique geographic region.

The endogenous proteinases, especially plasmin (PL), in bovine milk significantly affect milk quality during the storage and transportation (Annandarajah, Grewell, Talbert, Raman, & Clark, 2018; Yun & Imm, 2021). The proteolysis induced by the thermostable PL can result in the development of bitter flavor, increased viscosity, and the eventual formation of a gel during storage, limiting the shelf life and market potential of milk (Anema, 2019; Fan et al., 2024). Furthermore, the inactive zymogen of PL, plasminogen (PLG), is a significant component of the potential PL activity and can be activated by two families of plasminogen activators (PAs), urokinase-type PA (uPA) and tissue-type PA (tPA) (Rauh et al., 2014). The activity of PL is influenced by various factors, including the stage of lactation, breed, cow's age, and the presence of mastitis. However, there are little researches into the effect of altitude on PL activity. Understanding the PL activity of milk is essential for ensuring the quality and stability of milk during the transportation and storage, in consideration of the complex geographic environment of the Qinghai-Tibetan Plateau.

Therefore, the primary objective of this study was to investigate the effects of altitude, especially the high altitudes prevalent on the unique Qinghai-Tibetan Plateau, on milk quality, including milk production, composition and the activities of the PL enzyme system. This investigation offered a preliminary understanding of the prospective large-scale establishment of modern dairy farms introducing high-yield Holstein cows on the Qinghai-Tibetan Plateau.

## 2 Materials and methods

### 2.1 Materials

Urokinase and ε-aminocaproic acid (EACA) were purchased from Sigma-Aldrich Co., Ltd. (St. Louis, MO, USA). H-D-Val-Leu-Lys-pNA·2HCl (S-2251) was obtained from Beijing Asnail Biotechnology Co., Ltd. (Beijing, China). Trisodium citrate was acquired from Beijing Solarbio Science & Technology Co., Ltd. (Beijing, China), and gelatin was from Shanghai Aladdin Biochemical Technology Co., Ltd. (Shanghai, China). Tris-HCl was supplied by Beijing Biotopped Technology Co., Ltd. (Beijing, China). All the chemicals used in this study were of analytical grade.

### 2.2 Sampling

**Fig. 1:**
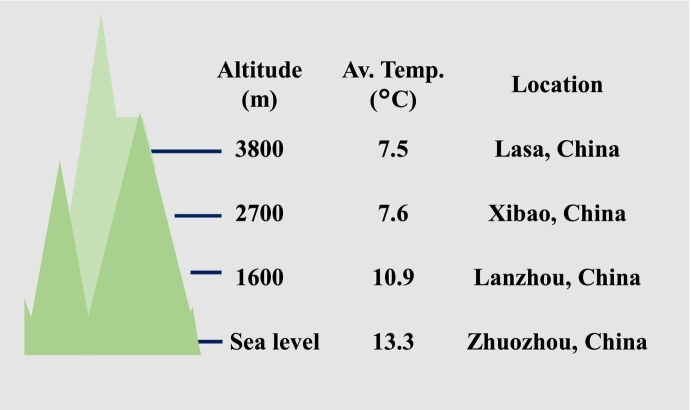
Sketch of the four pastures at varying altitudes sampled in this study.

Milk samples from Holstein cows were gathered from pastures at four distinct altitudes (Fig. 1): two high-altitude pastures at 2700 m (Xibao Village, Xining, China; annual average temperature of 7.6 °C) and 3800 m (Gaba Ecological Dairy Farm, Lasa, China; annual average temperature of 7.5 °C), a medium-altitude pasture at 1600 m (Lanzhou City, China; annual average temperature of 10.9 °C), and a low-altitude pasture ranging from 0 to 100 m (Zhuozhou Dairy Farm of China Agricultural University, China; annual average temperature of 13.3 °C). In each pasture, 30 cows were selected with a parity of 1, a lactation period of 100–130 days, and no signs of mastitis. Sampling was conducted in June, and a volume of 500 mL of milk was collected from each cow, totaling 15 L from each pasture. The feed formula was identical for all four pastures. After sampling, the 15 L of samples of milk were combined and stored in a 4 °C refrigerator for further analysis within 48 h.

### 2.3 Composition of raw milk

The protein, fat, lactose, ash, total milk solids, and somatic cell contents of the raw milk were measured in accordance with the standards of the People's Republic of China: GB 5009.5–2016, GB 5009.6–2016, GB 5009.8–2016, GB 5009.4–2016, GB 5413.39–2010, and NY/T 800–2004, respectively.

### 2.4 Determination activities of PL system

The method used to assess the PLG and PL activities in this study was adapted from Rauh et al. (Rauh et al., 2014). An aliquot (1 mL) of milk sample was mixed with 250 μL of 0.4 M tri‑sodium citrate buffer (pH 8.9) and agitated for 15 min to dissociate the casein micelles. The milk sample was then centrifuged at 4 °C for 10 min at 30,000 ×*g* to remove the fat. The skimmed milk sample was diluted (1:1 *v*/v) with buffer (0.1 M Tris-HCl, 8 mM EACA, 0.4 M NaCl, pH 8) and agitated for 15 min, to separate the PL and PLG from the casein. To determine the PLG-derived activity, the skimmed milk sample was diluted (1:1 v/v) with 2200 IU/mL urokinase buffer and agitated for 15 min, converting PLG to PL (Saint-Denis, Humbert, & Gaillard, 2001).The PL and PLG-derived activity were determined by measuring the hydrolysis rate of the chromogenic substrate S-2251, i.e., the subsequent release rate of p-nitroaniline. The sample solution was mixed with 4 mM S-2251 solution (1:1, v/v) and transferred to a 96-well plate, and incubated in a 37 °C water bath. The absorbance was recorded using a microplate reader (Infinite 200 Pro, Tecan, Austria) at both 405 nm and 490 nm with a measurement interval of 20 min, over six measurement time points. To correct the turbidity, the background absorbance (at 490 nm) was subtracted from the absorbance value at 405 nm. Linear regression was performed with time and absorbance values, and enzyme activity was then calculated based on a standard curve. The activity of PLG was determined as the difference between the activity of PL and that of the derived PLG. The total plasminogen activator (PA) activities, including the tPA and uPA activities, were detected with enzyme-linked immunoassay kits manufactured by Jiangsu Meimian Industrial Co., Ltd. (China).

### 2.5 Heat treatment

To investigate the effects of pasteurization on the activities of the PL derivative system (including PL, PLG, and PA), the raw milk was heated at 65 °C for 30 min in a water bath (DK-8B, Shanghai Jinghong Experimental Equipment Co., Ltd., China). The activities of PL, PLG, and total PA were determined according to the procedures reported in section 2.4.

### 2.6 Statistical analysis

All experimental data were represented as average value ± standard deviations. SPSS 18.0 data processing systems (SPSS Inc., USA) was used to perform the analysis of variance with Duncan's method. *p* < 0.05 indicated statistical significance.

## 3 Result and discussion

### 3.1 Composition of raw milk

The daily milk production (DMP), somatic cell numbers, and the composition of the raw milk are presented in Table 1. The somatic cell number is a crucial index used to monitor the occurrence of subclinical mastitis in herds or individual cows (Dufour & Dohoo, 2013). As shown in Table 1, there was no difference among four altitudes in the somatic cell numbers which were observed below 200,000 cells/mL, suggesting the Holstein cows were in good health with no mastitis or infection (Zecconi et al., 2020).

**Table 1:** Daily milk production and the composition of raw milk from various altitudes.

	Sea Level	1600 m	2700 m	3800 m
Daily Milk Production (kg)	33	26	17	9
Somatic cell number (10^4^/mL)	16.0 ± 0.85^a^	17. 2 ± 1.04^a^	17.6 ± 1.20^a^	16.2 ± 0.95^a^
Protein (%)	3.4 ± 0.01^b^	3.5 ± 0.03^b^	3.7 ± 0.09^a^	3.8 ± 0.11^a^
Fat (%)	3.7 ± 0.01^c^	3.8 ± 0.03^c^	3.9 ± 0.11^b^	4.3 ± 0.09^a^
Lactose (%)	5.0 ± 0.02^b^	5.1 ± 0.04^b^	5.2 ± 0.03^a^	5.1 ± 0.06^a^
Ash (%)	0.7 ± 0.04^b^	0.7 ± 0.04^b^	0.8 ± 0.05^a^	0.9 ± 0.03^a^
Total solids (%)	12.6 ± 0.03^c^	12.8 ± 0.07^c^	13.5 ± 0.08^b^	14.1 ± 0.12^a^

Values are average ± standard error (*n* = 15). Letters beside data in the same row indicate significant differences (*p* ≤ 0.05).

The obvious negative correlation between DMP and altitude could be observed from Table 1. The DMP decreased as the altitude increased, which might be attributed to the decrease in temperature and oxygen content at higher altitude (Mylostyvyi & Chernenko, 2019; Vaculikova et al., 2017). DMP is reported to correlate negatively with temperature within the range of −3.9 to 9.8 °C (Vaculikova et al., 2017). The cold stress significantly affects the daily milk yield, especially for high-yielding dairy cows. High-yielding dairy cows require a high intake of dry matter to support their levels of milk production (Hill & Wall, 2017). However, excessive cooling of the cow body can impair thermoregulation and fertility, induce cold stress, and reduce milk production, even with increased feed intake (Angrecka & Herbut, 2015). Furthermore, at high altitude, hypoxia increases the expensive anaerobic glycolytic pathways, reducing the metabolic availability of energy (Leiber et al., 2005). Therefore, the reduction in DMP could also be caused by the reduction in available energy associated with hypoxia. However, the DMP of the Holstein cows was >4-fold higher than that of yaks even at 3800 m, which showed a minimum value of 9 kg, confirming the feasibility of introducing Holstein cows on the Qinghai-Tibetan Plateau to enhance local milk production.

The components of raw milk, including protein, fat, lactose, ash, and total solids, increased significantly with altitude (Table 1). This was consistent with the observations in yak milk at higher altitudes, which showed significant increase in fat and total solids, together with a slight increase in proteins, lactose, and ash (Wu et al., 2009). However, Cui found there was no differences in the total solids or crude protein in milk at different altitudes (Cui et al., 2016). These may be attributed to the use of different forages at each altitude, which varied in several parameters, including biomass, protein, and neutral detergent fiber. In contrast, a constant feed formula was used in all four pastures in this study. it was reported that various changes in milk proteins, lactose, fat, total solids, and chlorides were observed as Holstein cows experienced high temperatures. Moreover, temperature fluctuations have been shown to significantly affect the composition of Holstein cow milk. Therefore, the differences in the composition of the milk in this study could be attributed to the distinct temperatures at different altitudes. Nevertheless, the precise mechanisms about the effect of low temperature on the composition of Holstein milk remain unknown due to the limited relevant research.

### 3.2 Activities of PL, PLG, and PA in raw milk

The PL, PLG, and PA activities and the PLG/PL ratios in raw milk at different altitudes are shown in Table 2. The activities of PL and PLG in this study were ranged from 0.28 to 0.59 mU/mL and 5.42–6.92 mU/mL, respectively, which were consistent with the acceptable ranges indicated by Rauh et al. (Rauh et al., 2014). However, the activity of PLG increased with increasing altitude, whereas the activity of PL decreased with increasing altitude, showing the different tendency with altitude. It has been reported that many factors including lactation number, stage of lactation, breed, mastitis, composition and quality of forage, and milking season affect the activity of PL and PLG (Leiber et al., 2005; Nicholas, Auldist, Molan, Stelwagen, & Prosser, 2002). Thus, these factors were controlled in this study, indicating that altitude was the predominant factor affecting enzyme activities. However, Christophe et al. reported an increase in PL activity at higher altitudes, whereas PLG activity remained independent of altitude (Christophe, Solange, Jean-Baptiste, Agnès, & Didier, 2001), contrary to our results. They believed that the increase in PL activity at high altitude was attributed to the changes in the composition of the vegetation and the extreme walking conditions for cows searching for food at high altitudes. In this study, the results could be considered to more reliable about the impact of altitude on PL activity as the identical artificial feed was used without additional natural grazing. The PLG/PL ratio ranged from 9.97 to 23.94, falling within the previously reported range from 2 to 30 for raw milk (Ismail & Nielsen, 2010). Both PA activity and PLG/PL ratio showed a consistent trend with PLG activity as the altitude increased. Interestingly, a clear distinction in the whole PL system could be observed at the critical elevation of 1600 m. The PL activity was notably lower in samples obtained from high altitudes (2700 and 3800 m) than in those from lower altitudes (sea level and 1600 m), even though their inactive zymogen (PLG) and activator (PA) displayed high activities. These results suggested that inhibitors within the PL system were present in higher concentrations at higher altitudes, thus inhibiting PL activity (France, O'Mahony, & Kelly, 2021).

**Table 2:** Activities of plasmin (PL), plasminogen (PLG), and plasminogen activator (PA) in raw milk from various altitudes.

	Sea Level	1600 m	2700 m	3800 m
PL (mU/mL)	0.52 ± 0.09^a^	0.59 ± 0.06^a^	0.28 ± 0.08^b^	0.38 ± 0.10^b^
PLG (mU/mL)	5.91 ± 0.42^b^	5.42 ± 0.35^b^	6.92 ± 1.22^a^	6.09 ± 0.36^a^
PA (mU/mL)	42.19 ± 5.08^b^	44.07 ± 4.14^b^	68.01 ± 8.15^a^	71.02 ± 9.60^a^
The ratio of PLG to PL	9.97 ± 1.92^b^	12.76 ± 2.12^b^	19.62 ± 6.47^a^	23.94 ± 8.11^a^

Values are means ± standard errors (*n* = 15). Letters beside data in the same row indicate significant differences (*p* ≤ 0.05).

### 3.3 Effect of heat treatment on the activities of PL, PLG, and PA

**Fig. 2:**
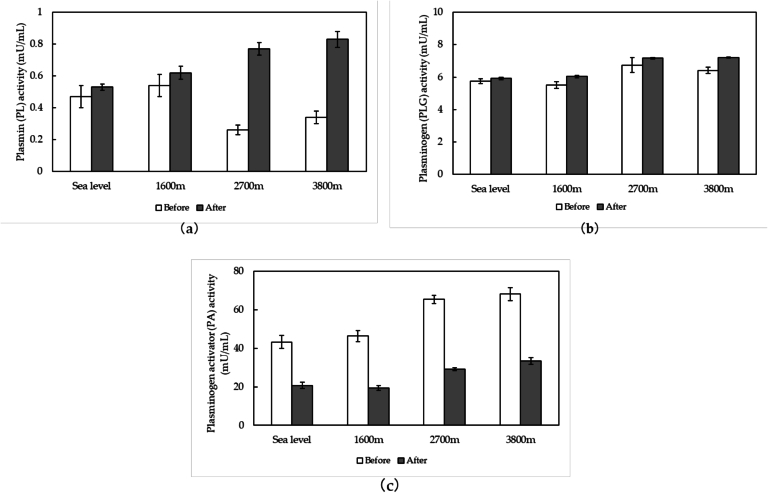
Activities of plasmin (a), plasminogen (b), and plasminogen activator (c) in milk from different altitudes before and after heat treatment (65 °C for 30 min). *n* = 15.

The effect of heat treatment (65 °C for 30 min) on the activities of PL, PLG, and PA is showed in Fig. 2. Across all the altitudes sampled, there was a slight increase in PLG activity and a significant reduction in PA activity after heat treatment. Furthermore, the milk samples obtained from high altitudes (2700 and 3800 m) showed significant increase in PL activity after being heated, whereas the samples from sea level and 1600 m showed only subtle increases in PL activity, which were not statistically significant. Thus, the pasteurization benefited the transportation and storage of milk collected at lower altitudes (sea level and 1600 m) due to the limited microbial activity and the inactivation of PA without significant change in PL activity. However, the PL activity of milk from high altitudes (2700 and 3800 m) dramatically increased after the pasteurization, resulting in the reduce in their shelf life.

The increase in PL activity after heat treatment was attributed to the conversion of PLG to PL, which could be caused by the partial thermal inactivation of inhibitors in the PL system (Chavan, Chavan, Khedkar, & Jana, 2011). An increase in the level of PL was also detected after the pasteurization and a short heat treatment (65 °C for 15 s) (Lu et al., 2009; Richardson, 1983). PL and PLG are reported to be more thermally stable enzymes than the inhibitors present in fresh milk, resulting in slight inactivation of PL and PLG after the pasteurization (Chavan et al., 2011). Consequently, the heat treatment of milk altered the natural balance between activators and inhibitors, enhancing the activity of PL. The increases in PL and PLG activities after heat treatment could also be related to the increased activation of denatured PLG (Ismail & Nielsen, 2010; Lu et al., 2009). During pasteurization, the denaturation of PLG caused the changes in tertiary structure. The unfolded kringles of PLG were more accessible to the action of PA, resulting higher PL and PLG activities. The activity of PA showed a significant decrease after pasteurization due to its partial thermal inactivation (Prado, Ismail, Ramos, & Hayes, 2007).

## 4 Conclusions

In this study, we investigated the impact of altitude on milk quality, including the DMP, milk composition, and the activities of the PL enzyme system. At higher altitudes, DMP was lower, with a minimum value of 9 kg below 3800 m. The protein, fat, lactose, ash, and total solid contents were higher in raw milk from high altitudes, showing higher nutritional value. The PL activity decreased with increasing elevation, whereas the PLG/PL ratio and the activities of PLG and PA increased with increasing altitude. The pasteurization significantly inactivated PA of milk collected from sea level and moderate altitudes (1600 m), favoring a longer shelf life. However, the pasteurization significantly increased PL activity of high-altitude milk samples, which could be problematic for the further transportation and storage of the milk. This study provided a theoretical reference to guide dairy-farming practices on the Qinghai-Tibetan Plateau by introducing high-yield Holstein cows to enhance local milk production.

## CRediT authorship contribution statement

**Wentao Qian:** Writing – review & editing, Writing – original draft, Methodology, Investigation, Data curation, Conceptualization. **Xiaobing Wang:** Writing – review & editing, Methodology, Data curation, Conceptualization. **Hongliang Li:** Writing – review & editing, Methodology, Investigation, Data curation. **Yinhua Zhu:** Writing – review & editing, Investigation, Data curation. **Pengjie Wang:** Writing – review & editing, Methodology, Funding acquisition, Data curation. **Xiaolu Geng:** Writing – review & editing, Investigation, Data curation. **Jinhui Yang:** Writing – review & editing, Methodology, Data curation. **Huiyuan Guo:** Investigation, Conceptualization. **Menghui Wang:** Methodology, Data curation. **Chong Chen:** Writing – review & editing, Project administration, Methodology, Data curation, Conceptualization.

## Declaration of competing interest

The authors declare that they have no competing interests.

## Data Availability

Data will be made available on request.
